# Isorhynchophylline Ameliorates Cerebral Ischemia/Reperfusion Injury by Inhibiting CX3CR1-Mediated Microglial Activation and Neuroinflammation

**DOI:** 10.3389/fphar.2021.574793

**Published:** 2021-02-12

**Authors:** Yuanyuan Deng, Ruirong Tan, Fei Li, Yuangui Liu, Jingshan Shi, Qihai Gong

**Affiliations:** ^1^ Key Laboratory of Basic Pharmacology of Ministry of Education, Department of Pharmacology, Zunyi Medical University, Zunyi, China; ^2^ Joint International Research Laboratory of Ethnomedicine of Ministry of Education, Zunyi Medical University, Zunyi, China; ^3^ International Center for Translational Chinese Medicine, Sichuan Academy of Chinese Medicine Sciences, Chengdu, China; ^4^ Department of Urology, Boston Children’s Hospital, and Harvard Medical School, Boston, MA, United States

**Keywords:** Isorhynchophylline, cerebral ischemia/reperfusion injury, neuroinflammation, microglia, C-X3-C motif chemokine receptor 1

## Abstract

Reperfusion therapy is an effective way to rescue cerebral ischemic injury, but this therapy also shows the detrimental risk of devastating disorders and death due to the possible inflammatory responses involved in the pathologies. Hence, the therapy of ischemia/reperfusion (I/R) injury is a great challenge currently. Isorhynchophylline (IRN), a tetracyclic oxindole alkaloid extracted from *Uncaria rhynchophylla*, has previously shown neuroprotective and anti-inflammatory effects in microglial cells. This study systematically investigates the effect of IRN on I/R injury and its underlying mechanism. The effects of IRN on neuronal injury and microglia-mediated inflammatory response were assessed on a rat model with middle cerebral artery occlusion (MCAO) and reperfusion-induced injury. We found that IRN treatment attenuated the infarct volume and improved the neurological function in I/R injury rats. IRN treatment also reduced the neuronal death rate, brain water content, and aquaporin-4 expression in the ischemic penumbra of I/R injury rats’ brains. Besides, IRN treatment could inhibit the following process, including IκB-α degradation, NF-κB p65 activation, and CX3CR1 expression, as well as the microglial activation and inflammatory response. These findings suggest that IRN is a promising candidate to treat the cerebral I/R injury via inhibiting microglia activation and neuroinflammation.

## Introduction

Stroke is the second leading cause of long-term morbidity and mortality worldwide (Pandian et al., 2018). Meanwhile, ischemic stroke accounts for more than 85% of all stroke cases, and ischemic stroke is usually caused by arterial blockage and followed by hypoxia and impaired glucose delivery to neuronal tissue. Restoration of the blood supply by using recombinant tissue plasminogen activator (tPA) can rescue the reversible damage penumbra of tissue and salvage the brain. However, the therapeutic time window of such thrombolysis agents is rigorously restricted to 4.5 h post-ischemic injury (Cronin et al., 2014). Meanwhile, reperfusion carries a wave of detrimental secondary injury risks. A high number of patients experience fatal edema or intracranial hemorrhage following thrombolysis. Reperfusion even causes a more extensive infarct in some cases (Chou et al., 2004; Khatri et al., 2012). This cerebral ischemia/reperfusion (I/R) injury is defined as the deterioration of the penumbra region, which is a salvageable brain tissue before reperfusion.

Inflammatory response occupies a vital role throughout the pathological course of the cerebral ischemia/reperfusion damage cascade. It is a dynamic process due to the abnormal activation of glial cells, especially the classically stimulated activation of microglia (M1). The M1 microglia generates pro-inflammatory cytokines such as TNFα and IL-1β, thereby forms a neuroinflammatory ischemic microenvironment that further aggravating neurological dysfunction, brain edema, and participating in the subsequent injury of the brain (Hu et al., 2012; Hu et al., 2015). Therefore, the anti-inflammatory agents are now drawing increasing attention to the application of cerebral I/R injury treatment.

*Uncaria rhynchophylla* (Miq.) (also named as Gouteng in Chinese), a Chinese herbal medicine, has been extensively used in formulas for centuries in the treatment of headache, epilepsy, and dementia (Shi et al., 2003; Fujiwara et al., 2006). The methanol extract of *Uncaria rhynchophylla* has shown a neuroprotective effect on cerebral ischemia-induced neuronal dysfunction by inhibiting the expression of COX-2 (cyclooxygenase-2) *in vivo* (Suk et al., 2002). Isorhynchophylline (IRN), a tetracyclic oxindole alkaloid segregated from *U. rhynchophylla*, has promising effects on animal models of Parkinson’s disease (Lu et al., 2012) or Alzheimer’s disease (Zhou and Zhou, 2012; Xian et al., 2014; Li et al., 2019). In addition, studies showed IRN and rhynchophylline (another valuable compound of *U. rhynchophylla)* suppressed the release of proinflammatory cytokines in LPS-induced microglial cells and inhibited the microglial activation (Yuan et al., 2009). that IRN has shown protective effect against the ischemia-induced neuronal damage in an *in vitro* study (Kang et al., 2004). However, there is a considerable lack of *in vivo* evidence of its role in ischemia-induced neuronal damage. Recent studies revealed that IRN can easily pass the blood-brain barrier (BBB) (Zhang et al., 2017; Zhang et al., 2019). These findings suggested that IRN could be an anti-inflammatory compound for the treatment of neurodegenerative diseases.

Given the effects of IRN on the inflammatory response (Yuan et al., 2009; Xian et al., 2019; Zhou et al., 2019), we speculate that IRN would reduce neuronal injury and microglia-mediated inflammatory response after I/R injury. In this study, a transient middle cerebral artery occlusion (MCAO) model followed with reperfusion was used to induce I/R injury, the effects of IRN on I/R injury as well as the possible underlying mechanisms of the effect of IRN were also explored in this MCAO model.

## Materials and Methods

### Reagent

Isorhynchophylline (IRN, purity  ≥  98%) was purchased from Nanjing Zelang Medical Technology Co., Ltd. All related reagents were of analytical grade and commercially available.

### Animals

Adult male Sprague-Dawley (SD) rats (3 months old; 250 to 300 g) were provided by the Experimental Animal Center of the Third Military Medical University (SPF-grade, Certificate No. SCXK2012-0011). Animal procedures were approved by the Animal Ethics Committee of Zunyi Medical University and conformed to National Institutes of Health Guide for Care and Use of Laboratory Animals.

### MCAO Model

Animals were anesthetized with sodium pentobarbital (50 mg/kg, i.p), followed by MCAO surgery as previously described which was developed by Longa et al. (Xing et al., 2008; Deng et al., 2016). Briefly, the common (CCA), external (ECA), and internal carotid artery (ICA) were exposed via a middle-line neck incision, and the connecting tissue was carefully dissected. A heat-blunted 30-mm nylon monofilament (diameter 0.22 to 0.24 mm) was inserted into the ECA and gently advanced 20 mm to block the blood flow of the right middle cerebral artery. The monofilament was withdrawn after 2 h occlusion of MCA. The sham groups were undergone the same procedure without the monofilament insertion. Blood pressure of animals during the surgery was monitoring from the tail with CODA Surgical Monitor. Body temperature was kept and monitored by placing animals on rectal probe-controlled heat pads of the entire procedures until they fully recover from anesthesia.

### Neurological Severity Scoring and Drug Treatment

Neurological severity scoring was done once a day after onset reperfusion by two inspectors not involved in this study, under the guidance of modified Bederson’s method: grade 0, no deficit; 1, reduced resistance to lateral push; 2, limb extension; 3, limb elevation; 4, unidirectional circling and decreased level of consciousness.

Rats that had bleeding complications during the surgery or after were excluded from the study. Around 30% percent of mice died after reperfusion 30 min to 48 h. The rest of sham rats and MCAO rats were randomly assigned to four groups, respectively: sham plus vehicle (sham + NS), sham plus IRN (sham + IRN), model plus vehicle (model + NS), and model plus IRN (model + IRN) groups. Drug treatment was initiated on the day after MCAO surgery. IRN was suspended in normal saline (NS), intragastrically administered 20 mg/kg once a day for three or seven consecutive days, the dosage of IRN was chosen based on previous pharmacodynamics studies (Xian et al., 2014; Zhang et al., 2019). The rats of sham and model vehicle-treated groups were administered with volume-matched of NS for the same duration. Animals were neurologically scored daily during the treatment period.

### Infarct Size Measurement

After 3 or 7 days onset of IRN treatment, animals were sacrificed after deep euthanized with sodium pentobarbital, and brain tissues were collected. two mm fresh brain slices were stained with 2% triphenyl tetrazolium chloride (TTC, Sigma) solution for 15 min at 37°C and fixed with cold 4% paraformaldehyde. Slices were photographed to calculate the infarct. Infarct volume was determined as the percentage of the contralateral hemisphere to correct for edema.

### Brain Water Content

Brain water content was detected using the standard wet/dry method. After dissecting the frontal pole, coronal brain slices were cut and divided into ipsilateral and contralateral hemispheres, wet weights were measured. Followed with drying slices in an oven at 100°C for 24 h to obtain the dry weights. Brain water content was calculated as a percentage: (wet weight–dry weight)/(wet weight) × 100%.

### Histology

Paraformaldehyde fixed and paraffin embedded tissues were coronally section on a Leica slicing machine to 4 μm for HE staining (Li et al., 2015b), Nissl staining (Liu et al., 2015), and Immunohistochemical staining (Deng et al., 2017) as previously described in detail. Primary antibodies against GFAP (1:500, Abcam) and IBA-1 (1:300, Abcam) were used in immunostained. Three digitized images were obtained from each section, and three sections were taken from each animal for quantitative analysis. The penumbra and ischemic core regions in cortex were chosen as areas of interest (AOIs).

### Western Blot

Protein extracts for Western blotting were prepared as previously described (Yin et al., 2018). Thirty μg protein of each sample was separated by SDS-PAGE followed by semi-dry transfer onto PVDF membranes. 5% non-fat milk were used to block the membranes and then incubated the membranes with the respective primary antibodies: aquaporin-4 (1:1,000), IL-1β (1:1,000), TNF-α (1:1,000), CX3CR1 (1:1,000), YM-1/2 (1:2,000) from Abcam company; p-NF-κB p65 (1:1,000), NF-κB p65 (1:1,000), IκB-α (1:1,000) from Cell Signaling Technology; and β-actin (1:5,000), GAPDH (1:5,000) from Beyotime Biological Technology. Blots were then visualized by HRP-coupled secondary antibodies (1:5,000, Beyotime) with an ECL detection kit (Beyotime) and digitized by Quantity One-4.6.7. (Bio-Rad).

### Statistical Analysis

Statistical analysis of results was executed with the Prism 8 software (GraphPad Software), two-way analysis of variance (ANOVA) and multivariate tests were used to evaluate neurological score among different groups. One-way ANOVA and Dunnett’s multiple comparison tests were used for statistical analysis of other data in this study. Data are expressed as mean ± SEM.

## Results

### IRN Reduced Brain Injury After Cerebral Ischemia

To induce the I/R injury, a transient middle cerebral artery occlusion (MCAO) surgery followed by reperfusion was performed on the rats. To evaluate the ischemia-reperfusion injury, the rats were sacrificed 3 or 7 days after IRN treatment, and the neurologic scores and infarct size were measured at the time points. As shown in Table 1, there was no neurological deficit observed in the sham group, while the I/R groups showed high scores during the periods. Compared with vehicle-treated MCAO group, a considerable improvement in neurological severity scoring can be seen after IRN 20 mg/kg treatment for 7 days (*p* < 0.05). However, no significant change was detected for 3 days of IRN administration (*p* > 0.05).

**TABLE 1:** Effect of IRN on neurological deficit.

Group	Treatment	Neurologic score			
		day1	day3	day5	day7
Sham	Saline	0	0	0	0
Sham + IRN	IRN 20 mg/kg	0	0	0	0
I/R	Saline	2.81 ± 0.16**	2.57 ± 0.17**	2.53 ± 0.17**	2.4 ± 0.16^**^
I/R + IRN	IRN 20 mg/kg	2.95 ± 0.16	2.35 ± 0.14	1.93 ± 0.07^**^	1.79 ± 0.11^**^

Values are mean ± SEM. n, 14 to 21. **P < 0.01 *vs* sham; ^##^ *P* < 0.01 *vs* NS.

**FIGURE 1:**
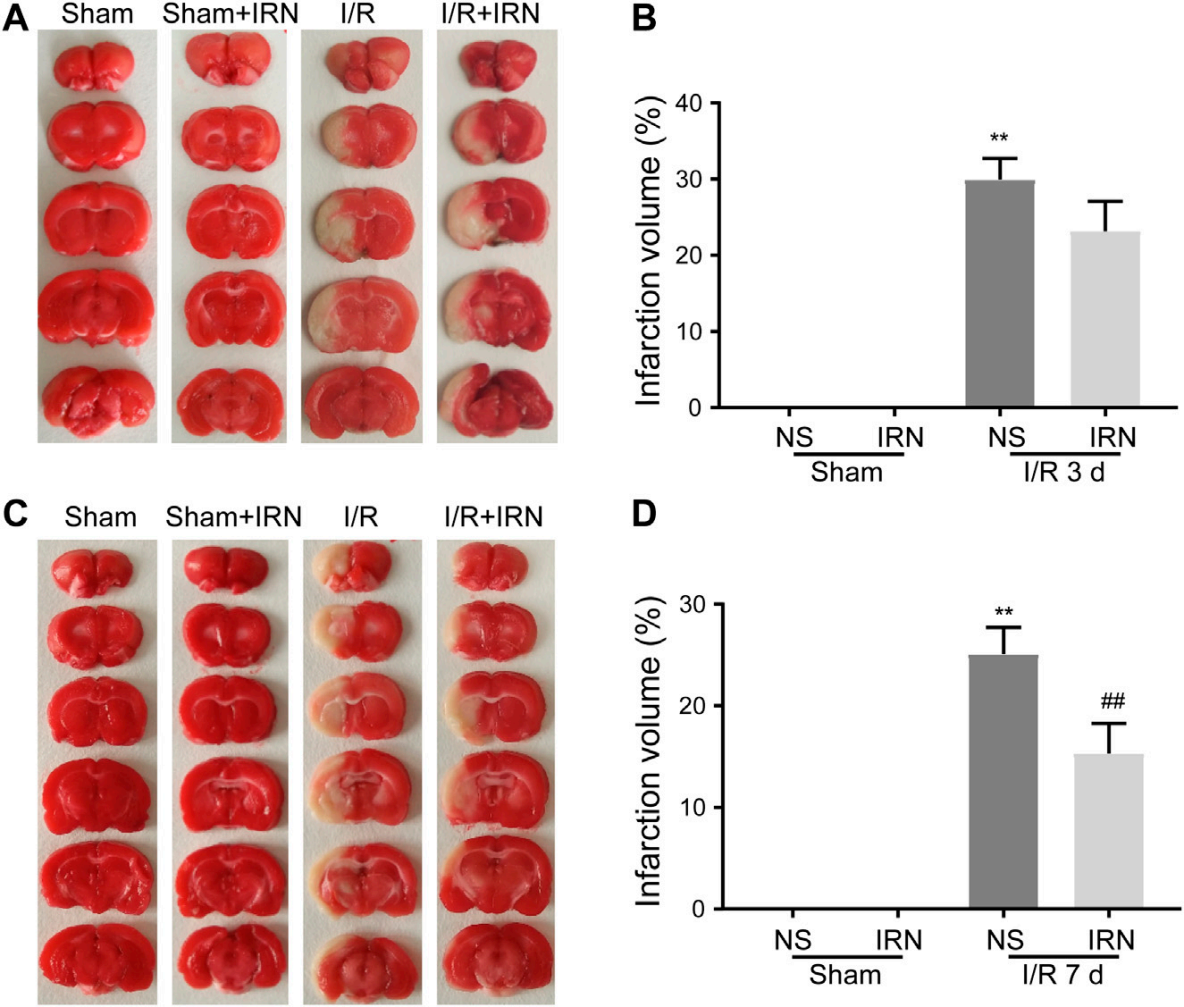
The IRN treatment diminished the infarct volume after I/R injury in rats. TTC staining assessed the infarct areas and infarct volume ratios after reperfusions for 3 days **(A)** and **(B)**, as well as 7 days **(C)** and **(D)**. Bars represent mean ± SEM of 4-6 brains. ^**^ *p* < 0.01 *vs* sham, ^##^ *p* < 0.01 *vs* NS.

To check the morphology change, the TTC staining was performed. From the brain sections, there was a significant difference between MCAO and sham animals. Extensive lesions can be seen in the sections of model group, with high infarct volumes in ischemia-reperfusion injury, in the group after 3 days or 7 days treatment of IRN. Compared with the MCAO group, the IRN treatment group showed a significantly smaller infarct volume at the time point of the 7^th^ day (*P* < 0.01, Figure 1). Thus, we focused on 7 days treatment of IRN, later on, to clarify the mechanism of IRN against cerebral I/R injury.

### IRN Prevented Neuronal Death After Cerebral Ischemia

**FIGURE 2:**
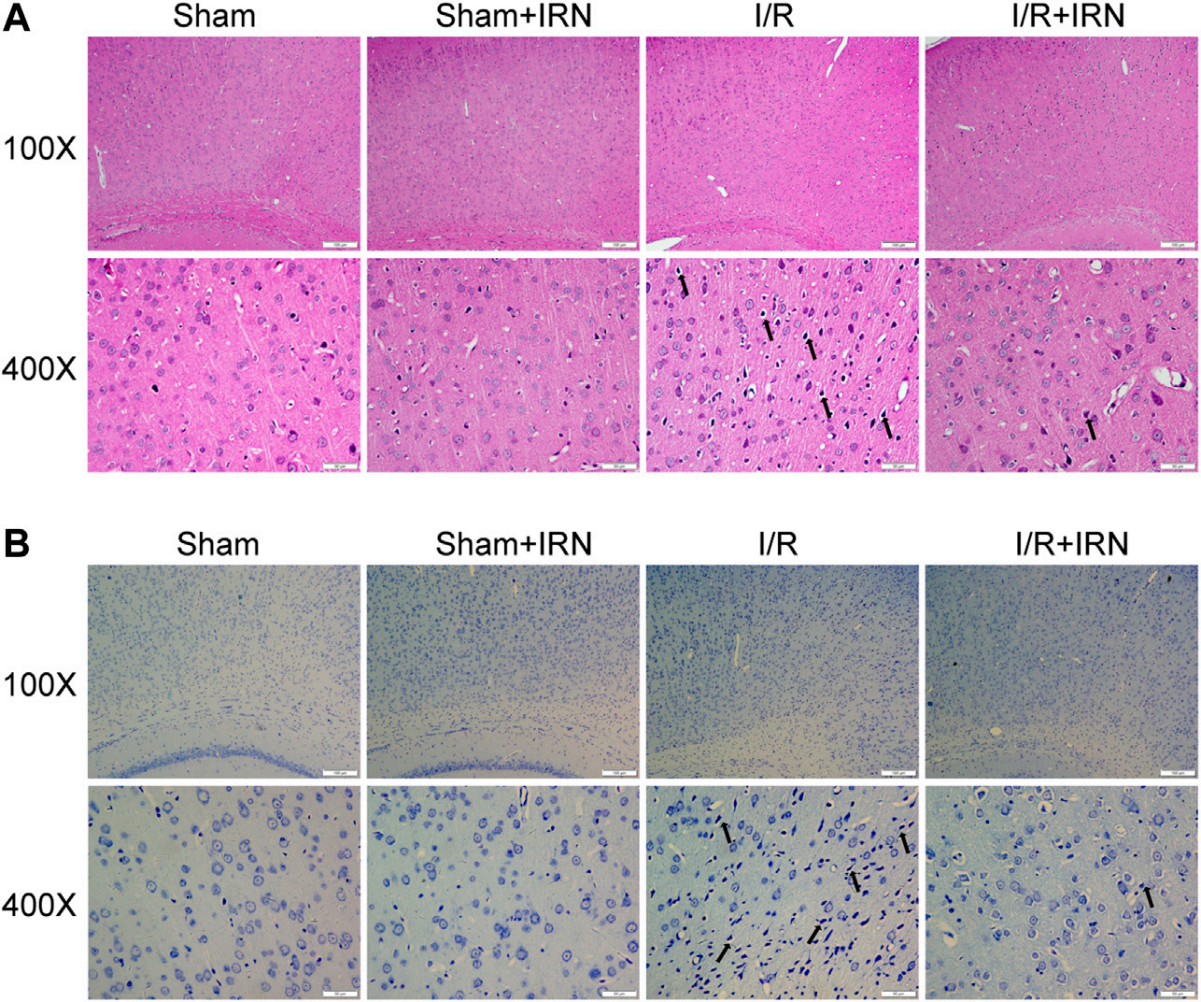
IRN treatment attenuated the morphological lesions after I/R injury in rats. The sections of penumbra region were obtained and undergone HE **(A)** and Nissl **(B)** staining. Magnification × 100 and 400 ×, scale bar = 200 or 50 μm.

We further investigated the effect of IRN on neuron survival by HE staining and Nissl staining. HE staining displayed normal arrangement and clear boundary for the neurons of ischemic penumbra region in Sham and Sham + IRN groups, while the MCAO rats exhibited vacuolization and pyknotic dead cells. After IRN treatment for 7 days, abnormal morphological alterations of neurons caused by ischemic stroke were alleviated and organized arrangement of the neurons can be seen. In Nissl staining, the Nissl bodies at ischemic penumbra of MCAO rats displayed atrophied morphology and disordered distribution compared to that of sham groups, while IRN remarkedly rescued the abnormal situation (Figure 2). Taken together, these results indicated that IRN could attenuate the morphological lesions caused by I/R injury.

### IRN Relieved Brain Edema After Cerebral Ischemia

**FIGURE 3:**
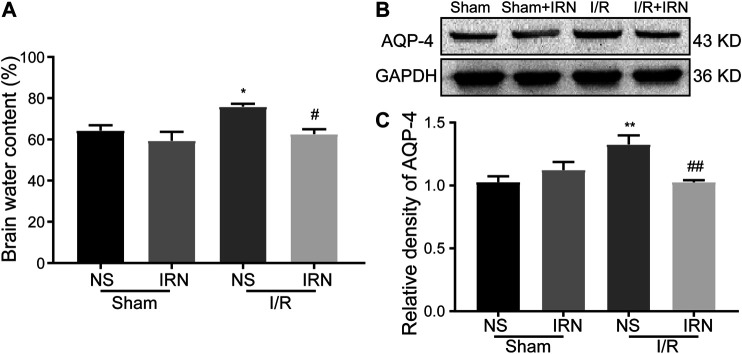
The IRN treatment relieved brain edema after I/R injury. Brain water content **(A)** measured by wet/dry method; The antibody-reactive bands **(B)** and relative density **(C)** of AQP-4 protein. Values are mean ± SEM. n = 4. ^*^ *P* < 0.05 and ^**^ *P* < 0.01 *vs* sham; ^*#*^ *P* < 0.05 and ^##^ *P* < 0.01 *vs* NS.

As the degree of brain edema is associated with the clinical indications after ischemia-reperfusion injury, the brain water content, and the expression of aquaporin-4 (AQP-4) were also detected. The brain water content in the I/R injury animals was significantly higher compared to that of the sham group (Figure 3A, *p* < 0.05), and the expression of AQP-4 was also increased after 7 days reperfusion (Figure 3B, *p* < 0.05). Also, reduced brain water content and decreased AQP-4 expression in ischemic penumbra were observed in IRN treated I/R injury animals compared to NS-treatment.

### IRN suppressed the activation of inflammatory cells induced by I/R injury

**FIGURE 4:**
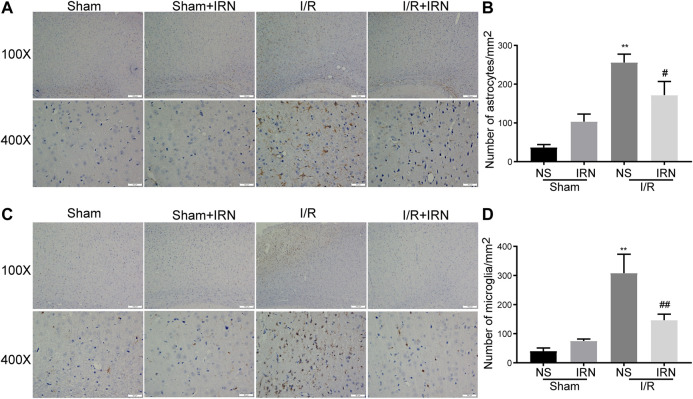
The IRN treatment suppressed the activation of astrocytes and microglia after I/R injury. GFAP immunoreactivity **(A)** and the number of astrocytes **(B)** in cortex penumbra region. IBA-1 immunoreactivity **(C)** and the number of microglia **(D)** in cortex penumbra region. Magnification × 100 and 400 ×, scale bar = 200 or 50 μm. Data were expressed as mean ± SEM, n = 5. **P < 0.01 vs sham; ^#^ *P* < 0.05, ^##^ *P* < 0.01 vs NS.

The neuroinflammatory response has been validated to produce a secondary injury after ischemia/reperfusion (Shah et al., 2009; Lee et al., 2014). The activation of inflammatory cells astrocytes and microglia was examined by assessing the expression of GFAP and IBA-1 through immunohistochemistry staining. Notable enhancements in both GFAP and IBA-1 expression in the penumbra cortex from I/R rats were observed as compared to sham group (Figure 4, *p* < 0.01, *p* < 0.01). Importantly, the number of positive-stained cells was decreased in the IRN-treated animals (*p* < 0.05, *p* < 0.01, respectively). These data indicated that IRN could suppress the inflammatory cells activation.

### IRN Attenuated Inflammatory Response and CX3CR1 Overexpression

**FIGURE 5:**
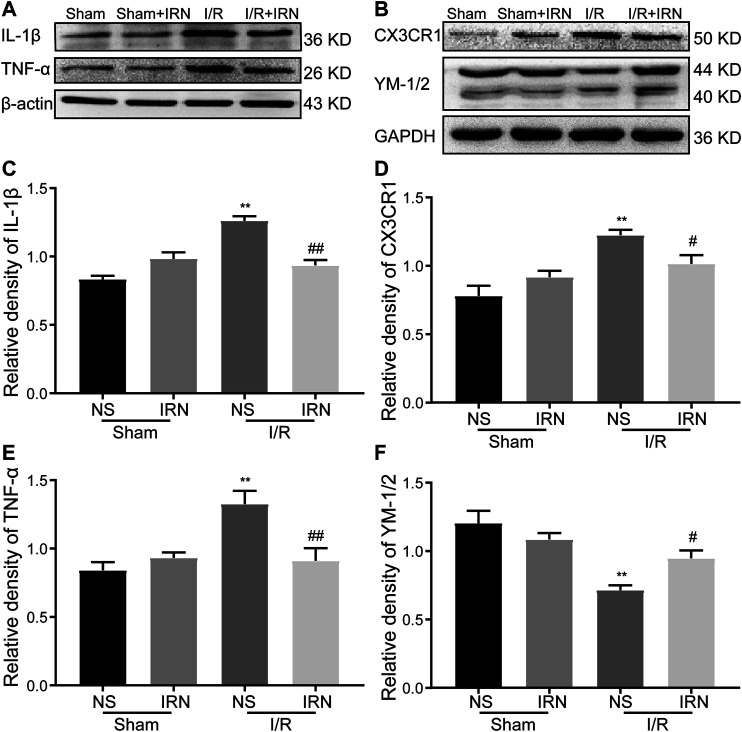
IRN altered the microglia phenotypes and regulated the protein expression of TNF-α, IL-1β, CX3CR1 and YM-1/2 after I/R injury. The antibody-reactive bands of TNF-α and IL-1β **(A)**, CX3CR1 and YM-1/2 **(B)**. Quantitative analysis of IL-1β **(C)**, CX3CR1 **(D)**, TNF-α **(E)** and YM-1/2 **(F)** in the penumbra cortex of different groups.. The relative optical density was normalized to β-actin or GAPDH. Data presented as mean ± SEM, n =4. **P < 0.01 vs sham; ^#^ *P* < 0.05, ^##^ *P* < 0.01 vs NS.

Following ischemia, activated microglia can potentially play either a protective or detrimental role. To determine the state of the activated microglia after MCAO, the expression of classical (proinflammatory, M1) and alternative (anti-inflammatory, M2) microglia markers were detected. There was a prominent induction in proinflammatory cytokines in the ischemic penumbra of the I/R group, while IRN treatment significantly suppressed these M1 markers expression, including TNFα and IL-1β (Figures 5A,C,E, *p* < 0.01, *p* < 0.01). In contrast, the expression of YM-1/2, which is an M2 marker, was decreased after MCAO compared to sham. However, the administration of IRN reversed the decreasing trend of YM-1/2(Figures 5B,F, *p* < 0.05), suggesting IRN might contribute to altering microglia phenotype to the M2 polarization in the brain of I/R-challenged rats. CX3C chemokine receptor 1 (CX3CR1) signaling has recently been found involved in the regulation of the interaction between neurons and resident microglia/migrated macrophages and the activation of microglia after I/R injury (Dénes et al., 2008; Tang et al., 2014). To further explore the mechanisms underlying the repression effect of IRN on microglia polarization, we next checked the expression of CX3CR1. CX3CR1 levels were notably increased after I/R compared with the sham animal, whereas IRN treatment for 7 days downregulated CX3CR1 expression levels (Figures 5B,D, *p* < 0.05).

### IRN Inhibited the Phosphorylation of NF-κB

**FIGURE 6:**
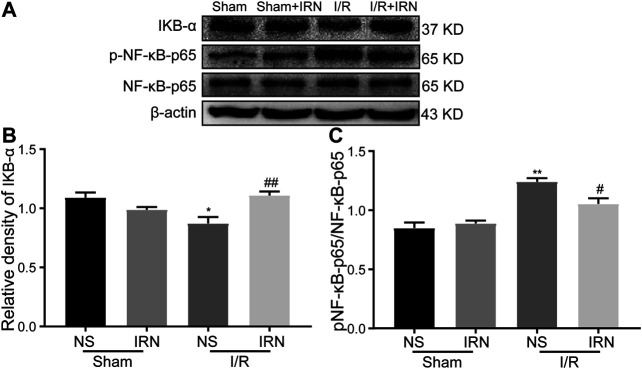
IRN rescued IκB-α degradation and restricted the NF-κB phosphorylation after I/R injury. The antibody-reactive bands of IκB-α and NF-κB p65 **(A)**. Quantitative analysis of IκB-α protein expression **(B)** and NF-κB p65 phosphorylation **(C)** in the penumbra cortex of different groups.. Values are mean ± SEM. n = 4. ^*^ *P* < 0.05 and ^**^ *P* < 0.01 *vs* sham; ^*#*^ *P* < 0.05 and ^##^ *P* < 0.01 *vs* NS.

The nuclear factor kappa B (NF-κB) is a key transcriptional factor involved in microglial activation and subsequent inflammation (Mattson and Camandola, 2001; Ridder and Schwaninger, 2009). As shown in Figure 6, decreased IκB-α expression and enhanced phosphorylation of NF-κB p65 were detected in I/R stimulated group compared to that of sham group (*p* < 0.05, *p* < 0.01). Importantly, IRN rescued IκB-α expression (*p* < 0.01) and limited the phosphorylation of NF-κB p65 protein after I/R injury when compared to that of sham group (*p* < 0.05). Collectively, these results indicated that IRN treatment ameliorated microglia-mediated inflammatory response via the NF-κB pathway during the I/R injury.

## Discussion

Cerebral I/R injury induced by transient suture occluded MCAO is a highly recommended experimental model to imitate the pathological evolution of human stroke (Xing et al., 2008; Boyko et al., 2010). In the current study, the neurologic grading scale and TTC staining showed high scores and large infarction areas in MCAO rats, which were consistent with the previous works of literature (Yang et al., 2013; Deng et al., 2016), indicating cerebral I/R injury model was successfully simulated. Notably, the present study portrayed, for the first time, that IRN had neuroprotection effects against ischemic injury/reperfusion (I/R) *in vivo* as 7-day-treatment of IRN decreased the infarct volume and improved the neurological function of I/R injury in rats, which is in line with the findings *in vitro* (Kang et al., 2004). However, the limitation of this study is the lack of data about the reduction in cerebral blood flow following MCAO, which can be achieved by Laser-Doppler flowmetry (LDF) (Kaundal and Sharma, 2011).

High death ratio and abnormal morphological alterations of neurons are hallmarks of I/R injury and have been the pivot of ischemia treatment (Zille et al., 2012). IRN 20 mg/kg displayed the ability to ameliorate the pathological outcomes of I/R injury in HE and Nissl staining. Brain edema is another detrimental hallmark of I/R injury as the increasing volume occupancy causes escalated intracranial pressure and even hernia (Görgülü et al., 2000; Zhu et al., 2013). The wet/dry method was applied to measure the brain water content, which is well recognized to evaluate the extent of brain edema. The brain water content was enhanced in model rats, while IRN treatment subsided the increase of water content. AQP4, the most abundant water channel in CNS, has a crucial role in maintaining brain water in equilibrium and responsible for clearing edema liquid under pathological conditions (Taniguchi et al., 2000; Papadopoulos and Verkman, 2007). Our results together with previous works of literature consistently illustrated that the expression of AQP4 increased along with the exacerbation of cerebral edema after I/R injury (Yao et al., 2015), suggesting brain edema initiated by I/R injury is associated with the process of water transportation, which involves AQP4. This study also displayed that IRN exerts neuroprotective effect via reducing the expression of AQP4, thereby attenuating the cerebral edema.

Inflammation is a consequence of cerebral I/R injury and promotes neuronal death and cerebral edema (Wang et al., 2007; Jin et al., 2010). Glial cells, especially microglia, are thought to be the primary responders and the principal immune cells that mediate neuroinflammation involved in I/R injury. Both the activation marker of astrocytes (GFAP) and microglia (IBA-1) were found up-regulated in the current model, whereas the number of GFAP and IBA-positive cells was reduced after IRN treatment. In fact, activated microglia have two phenotypes with opposite functions: M1 (classically activated) state, contribute to proinflammatory and neurotoxicity, secreting proinflammatory cytokines, including TNFα and IL-1β; while M2 (alternatively activated) state, has action on anti-inflammatory and exerts neuroprotective effects. Approaches that can shift microglia from the pro-inflammatory M1 to the anti-inflammatory M2 phenotype have been regarded as promising strategies to treat cerebral I/R injury. Since previous research showed that IRN could effectively alleviate the release of proinflammatory cytokines in N9 microglial cells induced by LPS (Yuan et al., 2009). We further looked inside the effects of IRN on the microglia polarization in I/R injury rats’ model. In the vehicle-treated I/R injury group, the expression of inflammatory factors, such as TNFα and IL-1β, was elevated after reperfusion of 7 days. Meanwhile, M2 hallmark YM-1/2 was decreased, which is in accordance with the previous studies (Pan et al., 2015). However, treatment with IRN restrained the increasing of M1 cytokines and optimized the expression of M2 hallmark, and then alleviated neuroinflammation response.

The NF-κB signaling pathway is pivotal in mediating inflammatory response after I/R injury. Normally, NF-kB is silent by binding to the inhibitory kB (IkB). Upon stimulation, like I/R injury, IkBs are rapidly phosphorylated and degraded, which enable NF-kB to be activated and eventually regulate this gene transcription. Recent evidence revealed that activated NF-kB stimulates CX3CL1 in neurons, and the enhanced CX3CL1 interacts with its receptor (CX3CR1), which is expressed on microglia, subsequently leads to the activation of microglia (Sheridan and Murphy, 2013; Li et al., 2015a). The current study also confirmed that NF-κB was activated and contributed to the neuroinflammation response through enhancing the expression of CX3CR1 during I/R injury. It is also worth mentioning that, the IκB-α degradation and the following phosphorylation of NF-κB p65, as well as the elevated CX3CR1 level, were limited after IRN treatment. Based on previous evidence which demonstrated the absence of CX3CR1 prevented the M1 activation microglia and attenuated ischemic injury (Fumagalli et al., 2013; Tang et al., 2014), our results indicated IRN suppressed NF-κB activation and CX3CR1 expression, consequently restrained the neurotoxicity activation of microglia, and eventually rescued the penumbra region from neuronal injury and cerebral edema.

In conclusion, this study demonstrated for the first time that IRN has neuroprotective effects on cerebral I/R injury *in vivo*. The underlying mechanisms may involve inhibition of the IκB-α degradation, NF-κB p65 activation, CX3CR1 expression, suppression of subsequent microglial activation and inflammatory response in the penumbra areas. Moreover, CX3CR1 deficiency animals need to be applied to confirm if microglia CX3CR1 is the direct target of IRN to exert its effects in the further study.

## Data Availability

The datasets used and/or analyzed during the current study are available from the corresponding author on reasonable request.
